# Small Signaling Peptides Regulate Rice Drought Stress Responses

**DOI:** 10.3390/plants15203100

**Published:** 2026-10-10

**Authors:** Zhijun Zhai, Wen Guo, Xifeng Wang, Min Xiong, Jing Wang, Cancan Fang, Min Chen, Meixia Wang

**Affiliations:** 1 Jiangxi Provincial Key Laboratory of Improved Variety Breeding and Efficient Utilization of Native Tree Species, Jiangxi Agricultural University, Nanchang 330045, China; 2 Jiangxi Key Laboratory for Excavation and Utilization of Agricultural Microorganisms, Jiangxi Agricultural University, Nanchang 330045, China; 3 Laboratory of Subtropical Biodiversity, Jiangxi Agricultural University, Nanchang 330045, China; 4 Shangrao Agricultural, Forestry and Water Sciences Research Center, Shangrao 334000, China; 5 School of Life Science, Nanchang Normal University, Nanchang 330032, China

**Keywords:** rice, small signaling peptides, drought stress, receptor-like kinase, genetic breeding

## Abstract

Drought stress is increasingly recognized as a major abiotic constraint on rice (*Oryza Sativa* L.) productivity and yield stability, posing a significant threat to global food security under rapidly changing and increasingly complex climatic conditions. To acclimate to water deficit, rice plants dynamically activate or suppress coordinated stress-perception systems, signal-transduction cascades, and multilayered regulatory networks. Over recent decades, extensive research has identified and characterized numerous drought-responsive genes, quantitative trait loci (QTLs), and drought-tolerant rice cultivars. Concurrently, small signaling peptides have emerged as pivotal regulatory molecules that orchestrate plant growth, development, and abiotic/biotic stress responses through binding with their cognate receptors. Nevertheless, the functional significance and underlying regulatory mechanisms of small signaling peptides in rice plant adaptation to drought stress remain insufficiently elucidated. To bridge this knowledge gap, this review summarizes recent progress in elucidating the roles and regulatory mechanisms of small signaling peptides in rice drought responses and outlines future research directions aimed at resolving outstanding unanswered issues and facilitating their application in the genetic development of water-efficient and drought-resilient rice cultivars.

## 1. Introduction

Drought represents one of the most significant environmental stressors that inhibit plant growth and development by restricting water absorption, modifying metabolic pathways, and disrupting physiological functions of RNAs, DNAs and proteins [1,2,3,4,5]. The increasing frequency and severity of drought events, exacerbated by climate change, have intensified the urgency for the development of resilient crop varieties capable of thriving under water-limited conditions. However, rice plants are highly susceptible to water scarcity. Drought stress not only impairs vegetative growth and reproductive development of rice plants but also disrupts essential physiological processes such as osmotic modifications, ultimately compromising rice performance and reducing grain yield [1,2,3,4,5]. Under water-deficit conditions, rice plants deploy a multifaceted suite of adaptive responses, encompassing the induction of reactive oxygen species (ROS) detoxification systems [6], remodeling of root system architecture [4], modulation of stomatal density and conductance [7], (de)activation of phytohormone signaling networks [5,8], epigenetic reprogramming [9], and transcription factor-mediated regulation of drought-responsive gene expression [10]. Collectively, these mechanisms optimize water acquisition efficiency and enhance drought stress resilience. Notably, developing water-saving and drought-tolerant rice cultivars through genetic improvement represents a more sustainable and feasible strategy for enhancing drought resilience while preserving yield potential. To this end, numerous rice cultivars have been developed, such as Huhan3, Yunlu99, and Hanyou73, which demonstrate remarkable yield performance under drought stress conditions [11,12]. Despite substantial advances in elucidating rice plant responses to drought stress over the past few decades [1,2,3,4,5,6,7,8,9,10,11,12], the molecular mechanisms underlying small signaling peptide-mediated drought responses in rice plants still remain largely unexplored.

Small signaling peptides, usually defined as being shorter than 100 amino acids in length, are a growing group of novel regulators that have been shown to play a key role in plant growth and environmental stress adaptation [13,14,15]. Plant small signaling peptides are typically classified into conventional peptides and non-conventional peptides (NCPs) [13,14,15,16,17]. Conventional peptides are sourced from well-defined coding regions or conventional open reading frames (ORFs) [13,14,15], whereas NCPs arise from untranslated or non-coding regions [16,17]. Additionally, small signaling peptides may be directly synthesized from short open reading frames (sORFs), which are found within the 5′ leader regions of mRNAs, primary transcripts of microRNAs (miRNAs), long non-coding RNAs (lncRNAs), ribosomal RNAs (rRNAs), circular RNAs (circRNAs), or transcripts encoding short proteins [14]. Generally, plant small signaling peptides can be further categorized into post-translationally modified (PTM) peptides, cysteine-rich peptides (CRPs), and peptides devoid of PTMs or CRPs that possess specific amino acid residues essential for their biological functions [13,14,15]. It is well known that plasma-membrane-localized receptors or co-receptors that belong to the leucine-rich repeat receptor-like kinases (LRR-RLKs) can perceive the conventional small signaling peptides, thus orchestrating plant growth and biotic/abiotic adaptation by (de)activating distinct downstream regulatory signaling cascades in various plant developmental and environmental adaptive processes [13,14,15].

To date, several classes of small signaling peptides have been identified in rice, including RAPID ALKALINIZATION FACTOR (RALF), CLAVATA3/EMBRYO-SURROUNDING REGION-RELATED (CLE), PLANT ELICITOR PEPTIDES (PEPs), CASPARIAN STRIP INTEGRITY FACTOR (CIF), C-TERMINALLY ENCODED PEPTIDE (CEP), microRNA-encoded peptides (miPEPs), IMMUNE RESPONSE PEPTIDE (IRP), phytosulfokine (PSK), and EPIDERMAL PATTERNING FACTOR (EPF)/EPF-LIKE (EPFL). These small signaling peptides mediate diverse physiological processes involved in rice growth and development, agronomic traits and in responses to abiotic and biotic stresses (Table 1).

In rice roots, OsRALF4 peptide maintains superoxide (O_2_^−^) homeostasis by suppressing THESEUS3–RBOHB signaling in the meristem and regulates pectin demethylesterification [18]. The resulting demethylesterified pectin subsequently associates with OsRALF4 peptide, promoting its assembly into compact bundles and thereby marking the onset of cell expansion [18]. OsRALF5 peptide functions through the rice FERONIA-LIKE RECEPTOR 1 (OsFLR1) to preserve the stem cell niche and root apical meristem (RAM) homeostasis, ultimately controlling root length [19]. Furthermore, the *osralf5* knockout mutants exhibited normal pollen viability but significantly impaired pollen germination and reduced seed set, indicating that OsRALF5 peptide, an atypical pollen-preferential RALF peptide, plays a critical role in pollen tube development [20]. OsRALF26 peptide is perceived by the OsFLR1 and XA21 receptors, leading to the induction of *pathogenesis-related* (*PR*) genes and ROS production and consequently conferring resistance to a subset of Xanthomonas oryzae pv. oryzae (*Xoo*) strains [21,22,23]. In addition, OsRALF17 and OsRALF19 peptides interact with rice MALE-GENE TRANSFER DEFECTIVE 2 (OsMTD2) receptor to regulate ROS homeostasis, thereby facilitating pollen tube germination and maintaining pollen tube integrity [24,25].

FLORAL ORGAN NUMBER 4 (FON4/FON2), FON2-LIKE CLE PROTEIN 1 (FCP1, also designated OsCLE402), and FON2 SPARE 1 (FOS1) peptides are close homologs of the Arabidopsis CLAVATA3 (CLV3) peptide and perform evolutionarily conserved functions in regulating the rice shoot apical meristem (SAM) and floral meristem (FM). FON4 peptide acts as a putative ligand for FLORAL ORGAN NUMBER (FON1), a CLAVATA 1 (CLV1) homologous receptor, thereby regulating SAM development [26,27,28]. Additionally, FON4 peptide modulates floral meristem (FM) activity through interactions with floral homeotic regulatory pathways, affecting spikelet number and highlighting its potential importance in increasing grain number and improving rice yield [29,30]. FON2-LIKE CLE PROTEIN1 (FCP1) and FCP2 peptides also contain CLE domains and are involved in negative regulation of meristem maintenance in the vegetative phase by regulating the expression of *WUSCHEL-RELATED HOMEOBOX4* (*WOX4*) [31]. Furthermore, FCP2 peptide inhibits root elongation and reduces root apical meristem (RAM) size by regulating the expression of the *QUIESCENT-CENTER-SPECIFIC HOMEOBOX* (*QHB*) gene [32]. The *FON2 SPARE1* (*FOS1*) gene also encodes a CLE peptide that acts along with FON2 peptide to maintain the FM [33].

Exogenous application of synthetic rice five-amino-acid peptide 1 (OsPEP1), as well as overexpression of *OsPEP1*, induces ROS accumulation in roots and consequently suppresses root elongation [34]. Application of synthetic OsPep5 peptide or constitutive overexpression of *OsPep5* reduces Na^+^ accumulation and lowers the Na^+^/K^+^ ratio in both shoots and roots by upregulating ion-transporter genes, thus improving salinity tolerance in rice [35]. OsCIF peptides cooperate with their putative receptor, SCHENGEN3 (OsSGN3), to regulate casparian strip (CS) formation by modulating the expression of *OsSHR1* and other CS-associated genes [36]. Treatment with a chemically synthesized 15-amino-acid OsCEP6.1 peptide leads to growth suppression in rice roots and seedlings, consistent with the phenotype observed upon *OsCEP6.1* overexpression. Furthermore, constitutive *OsCEP6.1* overexpression was sufficient to modify panicle architecture and grain size [37]. The *miR172b*-encoded peptide miPEP172b is a key regulator of salt stress tolerance in rice, coordinating ROS-scavenging mechanisms with phytohormone signaling pathways [38]. Additionally, miPEP156e peptide regulates cadmium tolerance and uptake through modulation of ROS signaling [39]. By contrast, the cysteine-rich peptide OsCDT3 directly binds aluminum, restricts its entry into root cells, and thereby enhances aluminum tolerance [40].

IMMUNE RESPONSE PEPTIDE 1 (IRP1) peptide strengthens resistance to the rice blast fungus *Magnaporthe oryzae* by inducing the defense-related gene *PHENYLALANINE AMMONIA-LYASE 1* (*PAL1*) and activating the MITOGEN-ACTIVATED PROTEIN KINASE (MAPK) signaling cascade [41,42]. The OsSSIP peptide, characterized by an N-terminal Tyr–Cys core motif, induces an ROS burst, MAPK phosphorylation, and the transcriptional activation of defense-related genes, thereby contributing to rice blast resistance [43]. Notably, rice PSKα peptide is perceived by the OsPSKR2 receptor, which promotes *PR* gene expression and ROS production, thereby enhancing blast resistance without compromising plant growth or grain yield [44]. It has also been reported that rice PEPs and PSK peptides exert antagonistic regulatory effects on wound-stress responses [45,46].

Perturbation of the rice EPF/EPFL members expression including *GRAIN NUMBER, GRAIN LENGTH AND AWN DEVELOPMENT 1* (*GAD1*) and *OsEPFL2* increases grain number per panicle while reducing grain length and promoting awn shortening or loss [47,48]. In addition, GAD1 and OsEPFL2 peptides facilitate seed germination by decreasing abscisic acid (ABA) levels and enhancing α-amylase activity and soluble-sugar mobilization during germination [49]. Furthermore, OsEPFL6, OsEPFL7, OsEPFL8, and OsEPFL9 peptides act synergistically to regulate rice panicle morphogenesis through recognition by the OsER1 receptor and subsequent activation of the MAPK signaling cascade [50].

In recent years, an increasing number of studies have elucidated the physiological functions of various small signaling peptides in the regulation of plant responses to drought stress. These small signaling peptides include CLE peptides [51,52,53], *Gossypium* Drought and Salt Resistance Peptide (GDRP) [54], CEP [55,56], EPF/EPFL [57,58,59,60], and PSK [61] peptides. These drought-responsive small signaling peptides regulate plant adaptation to water deficit by activating or repressing distinct signaling networks. Furthermore, the exogenous application of synthetic peptides or the overexpression of their corresponding encoding genes has been shown to confer significantly enhanced drought tolerance in several economically important crops and horticultural species, such as cotton (*Gossypium hirsutum* L.) [51,54], pepper (*Capsicum annuum* L.) [52], common bean (*Phaseolus vulgaris* L.) [53], tomato (*Solanum lycopersicum L.*) [55], wheat (*Triticum aestivum* L.) [56], potato (*Solanum tuberosum* L.) [59], maize (*Zea mays* L.) [60] and *Brassica napus* [61]. These findings highlight the considerable potential of manipulating small signaling peptide-encoding genes to develop drought-resilient crop and horticultural plants. Nevertheless, the functional characterization and application of small signaling peptides in the development of drought-tolerant rice cultivars currently remain limited.

This review aims to underline the pivotal biological functions of several currently characterized small signaling peptides, including OsDT11 [62], OsDSSR1 [63], OsEPF1 [7,64], OsRALF45 and OsRALF46 [65], OsCEP8 [66] and OsPSY8 [67], in orchestrating drought stress responses in rice plants. We further propose several directions for future research to address outstanding knowledge gaps and assess the potential risks associated with their application in agricultural and ecological systems. Collectively, precision genetic engineering of rice loci encoding drought-responsive small signaling peptides, together with the exogenous application of synthetic peptides, constitutes an innovative toolkit for developing drought-resilient rice cultivars and advancing sustainable agriculture.

**Table 1:** A summary of functional characterized small signaling peptides in rice.

Peptide Family	Peptide Name	Receptor	Downstream Signaling	Biological Function	Reference
RALF	OsRALF4	THESEUS3	RbohB and pectin demethylesterification	Root development	[18]
	OsRALF5	OsFLR1	-	RAM development	[19]
	OsRALF5	-	-	Pollen development	[20]
	OsRALF26	XA21/OsFLR1	ROS and *PR* genes	Defense response	[21,22]
	OsRALF17/19	OsMTD2	ROS	Pollen development	[24,25]
	OsRALF45/56		RbohA	Drought stress	[65]
CLE	FON4/FON2	FON1	-	SAM and FM development	[26,27,28]
	FON4	-	Floral homeotic genes	Flower development and spikelet meristem	[29,30]
	FCP1/FCP2	-	WOX4	SAM development	[31]
	FCP2	-	QHB	RAM development	[32]
	FOS1	-	-	FM development	[33]
CIF	OsCIF1/2	OsSGN3	OsSHR1 and other Casparian strip-associated genes	CS formation	[36]
CEP	OsCEP6.1	-	Cell cycle genes	Root and seedling development	[37]
	OsCEP8	-	ROS, auxin	Drought stress	[66]
miPEP	miPEP172b	-	ROS and phytohormone signaling	Salt tolerance	[38]
	miPEP156e	-	ROS	Cadmium tolerance	[39]
PSK	OsPSKα	OsPSKR2	ROS and *PR* gene	Defense response	[44]
EPF/EPFL	OsEPFL2/GAD1	-	-	Awn development and grain length	[47,48]
	OsEPFL2/GAD1	-	ABA and α-amylase	Seed germination	[49]
	OsEPFL6/7/8/9	OsER1	MAPK signaling	Rice panicle morphogenesis	[50]
	OsEPF1	-	-	Drought stress	[7,64]
**-**	OsPEP1	-	ROS	Root development	[34]
	OsPep5	-	Ion-transporter genes	Salt stress	[35]
	OsCDT3	-	-	Aluminum tolerance	[40]
	OsIRP1	-	*PAL1* and MAPK signaling	Defense response	[41,42]
	OsSSIP	-	ROS and MAPK signaling	Defense response	[43]
	OsDT11	-	ABA	Drought stress	[62]
	OsDSSR1	-	ROS and ABA	Drought stress	[63]
PSY	OsPSY8	-	ROS, lignin biosynthesis, compatible solute biosynthesis	Drought stress	[67]

Note: “-” means unknown.

## 2. Small Signaling Peptides Mediate Rice Drought Stress Response

### 2.1. OsDT11 Peptide Enhances Rice Drought Tolerance via ABA Pathway

**Figure 1:**
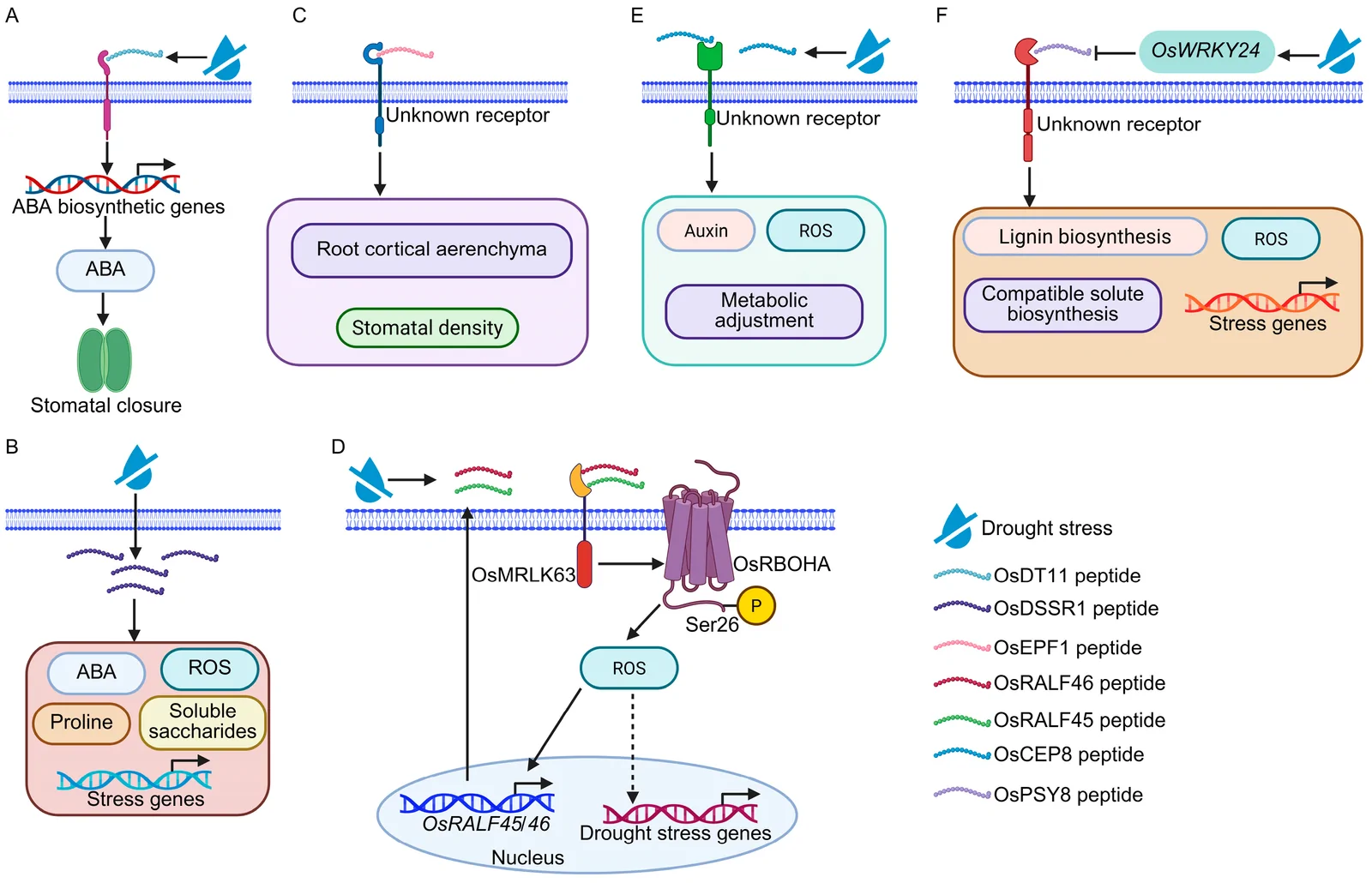
Small signaling peptides mediate the drought response in rice plants. (**A**) Drought conditions induce the expression of *OsDT11*, whereby an unidentified receptor facilitates the transduction of the OsDT11 peptide signal, thereby initiating ABA biosynthesis. The resultant accumulation of ABA subsequently leads to stomatal closure and enhances drought resilience. (**B**) Drought stress activates the biosynthesis of OsDSSR1 peptide via an unknown manner, the activated OsDSSR1 peptide then modulates ABA signaling pathways, as well as the accumulation of ROS, proline, and soluble sugars, thus contributing to drought stress responses. (**C**) The OsEPF1 peptide is perceived by as-yet-unidentified receptors, thereby modulating stomatal density and root cortical aerenchyma (RCA) to enhance drought tolerance. (**D**) The OsRALF45 and OsRALF46 peptides bind to receptor OsMRLK63, which in turn phosphorylates OsRBOHA at Ser26, leading to altered ROS levels. ROS then influence the expression of genes responsive to drought stress through yet-to-be-identified mechanisms. Additionally, ROS can promote the biosynthesis of OsRALF45 and OsRALF46 peptides, establishing a feedback regulatory loop in the drought response. (**E**) The OsCEP8 peptide modulates auxin signaling, ROS dynamics, and metabolic alterations to enhance drought tolerance in rice plants through an uncharacterized receptor. (**F**) Under drought stress conditions, *OsWRKY24* is induced and transcriptionally represses *OsPSY8* expression. OsPSY8 peptide may then be perceived by an as-yet-unidentified receptor, leading to the suppression of growth-associated transcriptional programs and the activation of stress-responsive programs. Dashed line means unknown mechanism. P: Phosphorylation.

*OsDT11* encodes an 88-amino-acid polypeptide with eight cysteines at the C-terminus, indicating that it is a member of the cysteine-rich peptides family [62]. OsDT11 peptide has a signal peptide at the N-terminal region that directs its localization to the cell wall. *OsDT11* expression is significantly induced by drought and salt stress, suggesting its biological roles in the rice abiotic stress response. Overexpression of *OsDT11* in rice plants leads to drought tolerance, as evidenced by a reduced wilting and lower water loss compared to that of wild-type plants. By contrast, *OsDT11* knockdown rice lines show increased drought sensitivity. Further investigation demonstrates that overexpression of *OsDT11* leads to an upregulation of abscisic acid (ABA) biosynthesis genes, resulting in increased ABA content (Figure 1A). The accumulated ABA thus triggers stomatal closure and confers drought tolerance in *OsDT11*-overexpressing rice plants (Figure 1A). A major limitation is that the putative receptors for the OsDT11 peptide and the precise drought-responsive signaling networks activated or suppressed by OsDT11 peptide remain to be elucidated.

### 2.2. OsDSSR1 Peptide Modulates Rice Drought Response via Antioxidant System

Rice *OsDSSR1* gene encodes a 75-amino acid polypeptide that is localized in both the nucleus and cytoplasm [63]. The transcriptional level of *OsDSSR1* in rice leaves is significantly induced by drought. Transgenic rice overexpressing *OsDSSR1* triggers an upregulation of antioxidant-related genes, increased the enzymatic activities of superoxide dismutase (SOD) and ascorbate peroxidase (APX), resulting in reduced ROS levels. RNA-seq analysis reveals significant changes in the expression level of many stress-responsive genes. In addition, elevated expression of *OsDSSR1* results in increased concentrations of free proline, ABA, and soluble saccharides. This accumulation of ABA and proline, together with the reduction in ROS levels, could promote rice tolerance to drought stress (Figure 1B). Classifying OsDSSR1 peptide as a secreted signaling peptide remains questionable, as its predicted N-terminal signal peptide has not been experimentally validated, and evidence for its apoplastic localization or interaction with a cell-surface receptor is lacking [63]. However, it is essential to comprehensively characterize the molecular and structural properties of the OsDSSR1 peptide and elucidate its specific biological functions and associated regulatory signaling cascades in responses to drought stress.

### 2.3. OsEPF1 Peptide Enhances Rice Drought Tolerance via Regulating Root Cortical Aerenchyma (RCA) and Stomatal Densities

Beyond the essential functions of OsEPF/OsEPFL peptides in awn development and seed germination [47,48,49], OsEPF1 peptide has also been implicated in drought stress responses [7,64]. Overexpression of *OsEPF1* markedly reduces stomatal density in rice plants [7,64]. This reduction is accompanied by increased stomatal size and altered stomatal conductance in *OsEPF1*-overexpressing rice plants. Collectively, these changes restrict leaf water loss, delay dehydration-induced leaf damage, and thereby enhance drought tolerance. Unexpectedly, root cortical aerenchyma (RCA), a specialized tissue characterized by extensive intercellular air spaces, develops constitutively in *OsEPF1*-overexpressing plants irrespective of tissue age or spatial position (Figure 1C) [64]. By contrast, *OsEPFL9* overexpression increases stomatal density, while *OsEPFL9* knockdown reduces stomatal density [64]. Notably, *OsEPFL9* overexpression does not affect rice plant performance under drought stress [64]. Reduced stomatal density entails a physiological trade-off. It lowers maximum stomatal conductance and may thereby restrict transpirational leaf cooling during heat events when soil moisture is sufficient [7]. Notably, under drought itself, low-stomatal-density rice remained cooler than wild type because conserved soil water sustained transpiration, indicating that the sign of the cooling trade-off depends on water availability. Because drought and heat stress frequently coincide under field conditions, these findings support optimizing rather than minimizing stomatal density and tailoring OsEPF peptide-mediated regulation to water-deficit conditions [7,64]. Further investigation is warranted to determine whether OsER1 or co-receptors such as TOO MANY MOUTHS (TMM) and SOMATIC EMBRYOGENESIS RECEPTOR-LIKE KINASE (SERK) [50] mediate OsEPF1 perception and the subsequent regulation of drought stress responses. Additionally, the precise molecular mechanisms underlying the OsEPF1 peptide-activated local or long-distance drought-responsive signaling network remain to be elucidated through in-depth investigation.

### 2.4. OsRALF45/46-OsMRLK63 Module Regulates Rice Drought Tolerance via Phosphorylation of ROS Production Enzyme

RALF peptides serve as pivotal modulators in plant development and stress acclimatization [68]. A total of 41 *OsRALF* genes have been identified in rice [24], which are crucial for root growth [18,19], immune responses [21,22,23], and the development of pollen tubes [20,24,25]. Notably, OsRALF45 and OsRALF46 peptides have been implicated in the response to drought stress [65]. Both OsRALF45 and OsRALF46 proteins are localized to the plasma membrane, and their expression levels are upregulated under drought conditions. The external application of synthetic OsRALF45 and OsRALF46 peptides has demonstrated the capability to improve rice performance under drought stress conditions. Further investigations reveal that OsRALF45 and OsRALF46 can physically interact with MALECTIN/MALECTIN-LIKE DOMAINS CONTAINING RECEPTOR-LIKE KINASE 63 (OsMRLK63) through its extracellular domain. The *osmrlk63* mutant rice exhibits insensitivity to OsRALF45 and OsRALF46 peptides, indicating that OsMRLK63 functions as the receptor for these peptides. Collectively, OsRALF45/46 peptides are synthesized as secretory prepropeptides that transit the plasma membrane via the default secretion pathway and are released into the apoplast, where they are recognized by the extracellular domain of OsMRLK63. Additional assays elucidate that OsMRLK63 interacts with and phosphorylates the ROS-producing enzyme, RESPIRATORY BURST OXIDASE HOMOLOGUE A (OsRBOHA), at Ser26 in the N-terminal region, which results in modifications to ROS levels and altered drought tolerance. Consequently, ROS may modulate the expression of drought-related genes via unknown mechanisms. Notably, ROS can also upregulate the expression of *OsRALF45* and *OsRALF46*, leading to their secretion into the apoplast where they are recognized by the OsMRLK63 receptor, implying a feedback regulatory mechanism governing the roles of OsRALF45/46 peptides in ROS generation and the drought stress response (Figure 1D). Nevertheless, whether and how other OsRALF family members participate in drought stress responses and associated regulatory cascades remain to be comprehensively elucidated.

### 2.5. OsCEP8 Peptide Modulates Drought Stress via Auxin, ROS and Metabolic Pathways

C-TERMINAL ENCODED PEPTIDES (CEPs) are classified within the family of post-translationally modified peptides, recognized for their involvement in various plant developmental processes such as systemic nitrogen (N) demand signaling, legume nodulation, shoot regeneration and the architecture of root systems as well as biotic and abiotic adaptations [69,70]. Specifically, CEPs have also been reported to regulate drought stress responses [55,56]. In wheat, TaCEP15 peptide binds to the TaCEPRL receptor, thereby enhancing the TaCEPRL-mediated phosphorylation of TaSnRK1α, which ultimately promotes its degradation, leading to the inhibition of primary root growth and increased sensitivity to drought stress. Additionally, TabZIP9 binding to the promoter region of *TaCEP15* results in reduced expression levels of *TaCEP15*. This alteration subsequently diminishes the ability of TaCEPRL to phosphorylate TaSnRK1α, impacting the protein stability of TaSnRK1α. Consequently, the elevated abundance of TaSnRK1α supports primary root development and improves drought resistance [56]. In rice, 15 *OsCEP* members have been identified, with *OsCEP8* exhibiting the most significant expression changes under abiotic stress conditions [66,71]. Transgenic rice lines overexpressing *OsCEP8* demonstrate enhanced drought resilience, characterized by reduced electrolyte leakage and ROS levels, as well as increased relative water content (RWC) and photosynthetic efficiency. Moreover, the overexpression of *OsCEP8* induces the downregulation of auxin conjugation-related genes, resulting in auxin accumulation and the potential promotion of growth during drought stress. Additionally, the OsCEP8 peptide is implicated in the activation of KIN10/SnRK1 signaling pathway genes, including *bZIP*s, resulting in the modulation of energy metabolism, catabolic processes and metabolic adjustment [66] (Figure 1E). Taken together, OsCEP8 peptide modulates drought stress adaptations via distinct cellular mechanisms. Notably, the cognate receptors of the OsCEP8 peptide and the upstream–downstream regulatory hierarchy of the multiple signaling pathways underlying OsCEP8 peptide-mediated drought adaptation warrant further comprehensive investigation.

### 2.6. OsPSY8 Peptide Negatively Regulated Rice Drought Stress Response

PLANT PEPTIDES CONTAINING SULFATED TYROSINE (PSY) are post-translationally modified signaling peptides whose precursors require tyrosine sulfation for full biological activity. Eight *OsPSY* genes have been identified in the rice genome, several of which show drought-responsive expression [67]. Among them, *OsPSY5* and *OsPSY8* are predominantly expressed in roots, and their transcript levels are downregulated markedly under drought stress, implicating both genes in the drought response [67]. Consistent with this expression pattern, *OsPSY8*-overexpressing rice plants exhibit greater cellular damage under drought stress, whereas the *OsPSY8* loss-of-function rice mutant displays enhanced osmotic-stress tolerance. By contrast, *OsPSY5*-overexpressing plants do not differ significantly from wild-type rice plants, suggesting a more prominent role for *OsPSY8* in drought responses [67]. Further investigations demonstrate that drought stress markedly activates *OsWRKY24*, which then functions as a direct repressor of *OsPSY8*. Transcriptomic analyses show that *OsPSY8* overexpression and loss-of-function rice plants elicit relatively specific changes in gene expression under drought stress. The differentially expressed genes are enriched in pathways associated with lignin biosynthesis, ROS scavenging, compatible solute biosynthesis, and stress responses (Figure 1F). Collectively, these findings suggest that constitutive *OsPSY8* expression suppresses stress-responsive pathways while favoring growth-associated transcriptional programs under drought stress conditions, whereas loss of *OsPSY8* function activates stress-responsive pathways [67]. Notably, the potential involvement of the cognate PSY receptor (PSYR) in the OsPSY8 peptide-mediated trade-off between growth and stress responses warrants further investigation.

## 3. Future Perspectives

As a consequence of accelerated climate change, numerous regions across the globe are anticipated to face severe drought conditions in the future [72,73]. Rice is a major staple crop worldwide, with global production projected to reach 2.0 × 10^9^ t by 2030 [74]. However, rice is highly sensitive to drought stress, which can substantially impair growth and reduce grain yield [74]. Although numerous genes involved in drought adaptations have been identified in rice plants [1,2,3,4,5,6,7,8,9,10], the functional roles of small signaling peptides in regulating rice drought stress responses remain largely unexplored. In recent years, plant-derived small signaling peptides have emerged as intrinsic regulators of plant growth and adaptive responses to environmental stress. Their biodegradability and biocompatibility make them promising alternatives for agricultural applications [75]. Consequently, OsDT11, OsDSSR1, OsEPF1, OsRALF45/OsRALF46, OsCEP8 and OsPSY8 peptides [62,63,64,65,66,67] represent valuable genetic resources for enhancing drought tolerance in rice breeding. Nevertheless, several fundamental questions remain to be addressed in future studies.

### 3.1. Identification of Unexplored Drought Associated Small Signaling Peptides in Rice

**Figure 2:**
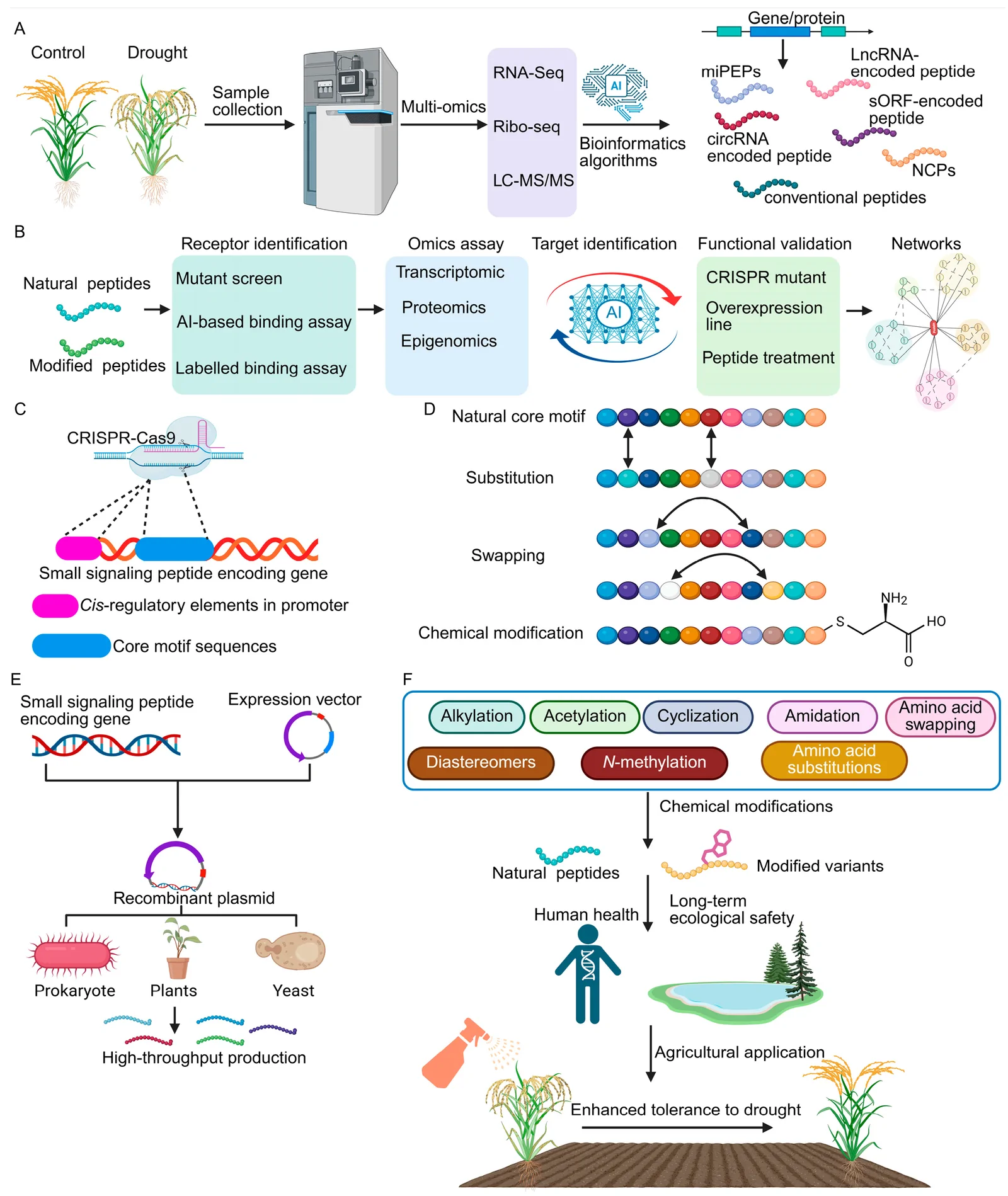
Identification and application of drought-responsive small signaling peptides in agricultural practice. (**A**) Employing AI-driven predictive models and advanced bioinformatics algorithms to integrate transcriptomic, LC-MS/MS, and ribosome profiling data will facilitate the discovery of previously uncharacterized rice drought-responsive small signaling peptides. (**B**) An illustrative schematic depicting the methodologies for identifying potential receptors and establishing drought-responsive signaling networks mediated by small signaling peptides in rice. (**C**) CRISPR–Cas9-mediated engineering of promoter regions and core motif sequences in genes encoding drought-responsive small signaling peptides to enhance transcriptional output and peptide bioactivity. (**D**) Rational redesigning the core motif sequences via amino acid substitution and swapping, or chemical derivatization to enhance their bioactivity. The colors represent different amino acid residues. (**E**) Synthetic biology-guided optimization of prokaryotic and eukaryotic expression platforms for the high-throughput, cost-effective production of bioactive, rice drought stress-responsive small signaling peptides in rice. (**F**) Safety and log-term assessment of drought-responsive small signaling peptides to facilitate their application in enhancing drought tolerance in rice.

Over the past few decades, diverse approaches including bioassay-guided screening, forward genetic screening, bioinformatic prediction, and multi-omics analyses have been successfully employed to identify plant small signaling peptides [75]. Advanced mass spectrometry (MS)-based peptidomics methodologies have enabled more sensitive and efficient identification of low-abundance endogenous signaling peptides [75]. Integrated transcriptomic (RNA-seq), proteomics, and ribosome profiling (Ribo-seq) analyses have identified small signaling peptides in rice that are involved in salt stress responses [35] and immune regulation [41]. Additionally, several machine learning algorithmic frameworks such as ExamPle and SE-SSP have been developed for the investigation of plant small signaling peptides [76,77], which will facilitate the systematic discovery of novel small signaling peptide families through integrative computational analysis. Consequently, the synergistic application of RNA-seq, MS, Ribo-seq, and advanced artificial intelligence (AI)-based mathematical modeling can be harnessed to uncover previously uncharacterized drought-responsive small signaling peptides including the non-conventional peptides (NCPs) in rice plants (Figure 2A).

### 3.2. Discovery of Adaptive Phenotypes Programmed by Drought-Responsive Small Signaling Peptides

Drought-adaptive phenotypes encompass the morphological adjustments that enable plants to survive under water-deficit conditions. These changes include root system architectural remodeling, reduced leaf expansion and cell proliferation, leaf rolling, and stomatal density and closure [3,4,7,8]. The CLE peptides have been shown to act as local or long-distance signaling to regulate stomatal closure and lateral root development under drought stress conditions [53,78,79,80,81]. In rice, OsEPF1 peptide regulates stomatal density and RCA under drought stress [7,64]. In addition, wheat TaCEP15 peptide modulate drought responses by regulating lateral root development [56]. These findings indicate that drought-responsive small signaling peptides promote drought tolerance by modulating plant morphology. The molecular mechanisms by which rice plants transduce signals from drought-responsive small signaling peptides into diverse adaptive morphological phenotypes, and the specific morphological adaptations underlying enhanced drought tolerance, however, remain to be elucidated.

### 3.3. Building the Drought-Responsive Regulatory Signaling Networks

As illustrated in Figure 1, the precise drought adaptation mechanisms mediated by OsDT11, OsDSSR1, OsEPF1, OsRALF45/OsRALF46, OsCEP8 and OsPSY8 peptides in rice plants remain largely elusive. The response of rice plants to drought stress involves the activation and deactivation of various cellular mechanisms, encompassing (post)transcriptional regulation, (post)translational modifications such as (de)phosphorylation, ubiquitination, and SUMOylation, as well as epigenetic regulation [1,2,3,4,5,6,7,8,9,10]. Recently, advanced sequencing techniques, including single-cell RNA sequencing and proteomics, have been employed in rice research to clarify the molecular underpinnings of environmental responses and developmental processes [82,83,84]. Furthermore, CRISPR-based epigenetic systems, alongside epigenetic sequencing methodologies such as MeRIP, m^6^A-LAIC-seq, CUT&Tag, and ATAC-seq, offer a comprehensive toolkit for epigenetic modulation via histone modifications, DNA/RNA methylation, and the regulation of genes encoding non-coding RNAs [85,86]. Consequently, the integration of these advanced multi-omics approaches is likely to facilitate the elucidation of unprecedented (post)transcriptional and (post)translational as well as epigenetic networks orchestrated by OsDT11, OsDSSR1, OsEPF1, OsRALF45/OsRALF46, OsCEP8 and OsPSY8 peptides in rice plants under drought stress conditions (Figure 2B).

Importantly, receptor-like kinases (RLKs) serve as critical hubs for the transduction of excretory small signaling peptide signals [13,14,15]. Notably, the cognate receptors responsible for perceiving the OsDT11, OsDSSR1, OsEPF1, OsRALF45/OsRALF46, OsCEP8 and OsPSY8 peptides under drought stress conditions remain to be elucidated. Accordingly, it is imperative to elucidate the corresponding receptors for drought-responsive small signaling peptides in rice through a variety of approaches, for instance, 4-azidosalicylic acid (ASA)-conjugated peptides and CRISPR-mediated genetic screening techniques [14,87]. The putative receptors can also be identified through AI-driven virtual binding screening that leverages RLK structural database [88], with subsequent validation conducted via a series of binding assays [89].

### 3.4. Interactions Among Drought-Responsive Small Signaling Peptides and Phytohormones

To date, more than 30 families of small signaling peptides have been identified in plants [13], and only 6 types of small signaling peptides have so far been functionally characterized in rice drought stress adaptations (Table 1; Figure 1) [62,63,64,65,66,67]. The CLE peptides are also implicated in the drought response [51,52,53,78,79,80,81]. More than 40 *CLE* genes have been identified in rice [90,91]. The functional role of CLE peptides in the context of drought stress, however, remains largely uninvestigated. Additionally, 11 *OsEPF*/*EPFL* [48], 41 *OsRALF* [24] and 15 *OsCEP* [37,71] and 8 OsPSY [67] genes have been identified in rice. Notably, only OsEPF1 [64], OsRALF45/OsRALF46 [65], OsCEP8 [66] and OsPSY8 [67] peptides have been shown to be involved in rice drought stress response; therefore, it would be valuable to investigate whether additional members of the rice CLE, RALF, EPF/EPFL, PSY and CEP families participate in the regulation of drought stress responses. Therefore, systematic and comprehensive investigations are required to determine whether and through which molecular mechanisms the OsDT11, OsDSSR1, OsEPF1, OsRALF45/OsRALF46, OsCEP8 and OsPSY8 peptides interact synergistically or antagonistically with as-yet-uncharacterized candidate small signaling peptides, such as members of the CLE family.

Evidence suggests that OsDT11, OsDSSR1, OsRALF45/OsRALF46, OsCEP8, and OsPSY8 peptides in rice converge on shared signaling pathways, for instance, ROS and ABA (Figure 1), indicating potential uncharacterized interaction nodes among these small signaling peptides under drought stress conditions. Furthermore, plant hormones, including abscisic acid (ABA), auxin, jasmonic acid (JA), and ethylene, are key regulators of drought stress responses in rice [5,8]. In addition, Ca^2+^, osmoprotectants such as proline, glycine betaine, and soluble sugars, as well as γ-aminobutyric acid, flavonoids, and melatonin, also contribute to drought stress responses. A pertinent question remains regarding how rice plants intricately regulate the complex interactions between OsDT11, OsDSSR1, OsEPF1, OsRALF45/OsRALF46, OsCEP8 and OsPSY8 peptides and phytohormones, ROS, Ca^2+^, osmoprotectants, γ-aminobutyric acid, flavonoids, and melatonin to optimize their growth and physiological performance under drought stress.

### 3.5. Genetic Manipulation and Redesigning of Rice Small Signaling Peptides to Enhance Drought Tolerance

Genetic modification enables stable phenotypic outcomes through targeted alterations of genomic information [92]. Therefore, *OsDT11*, *OsDSSR1*, *OsEPF1*, *OsRALF45*/*OsRALF46*, *OsCEP8* and *OsPSY8* genes represent promising molecular targets for marker-assisted selection and genome-editing-based breeding. However, the quantitative contributions of individual loci to drought resilience across diverse rice genetic backgrounds remain to be systematically elucidated. Promoter engineering via multiple *cis*-regulatory elements (CREs) has emerged as an effective strategy for modulating rice architecture, grain quality, and stress resistance [92]. In maize, CRISPR–Cas9-mediated editing of *CLE* gene promoters altered *CLE* transcriptional activity and enhanced grain yield [93]. This approach may therefore be applicable to the precise modulation of *OsDT11*, *OsDSSR1*, *OsEPF1*, *OsRALF45*/*OsRALF46*, *OsCEP8* and *OsPSY8* promoters, thus manipulating their expression and improving drought tolerance in rice plants under drought conditions (Figure 2C).

Exogenous application of synthetic peptides harboring key sequence motifs can recapitulate the biological functions of their endogenous counterparts [13,14,15]. Accumulating evidence highlights the potential of engineering bioactive core motifs derived from small signaling peptides [94]. Such peptide analogs can be systematically optimized through strategies including amino acid substitution and swapping, or chemical derivatization, which may result in greater biological efficacy than that of their native forms [94]. Hence, rational engineering of key motifs within protein sequences can generate variants with enhanced transcriptional activity and increased bioactivity of the corresponding small signaling peptides (Figure 2D) [94]. Nevertheless, excessive accumulation of small signaling peptides may inhibit plant growth [13,14,15]; thus, their expression levels must be carefully optimized to balance drought stress resilience with normal growth and development in rice plants.

Diverse chemical modifications including alkylation, acetylation, cyclization, incorporation of diastereomeric residues, amidation, and N-methylation, have been demonstrated to modulate peptide stability and biological functionality [95]. However, their effects on the bioactivity of OsDT11, OsDSSR1, OsEPF1, OsRALF45/OsRALF46, OsCEP8 and OsPSY8 peptides remain largely unresolved. Further investigation is therefore warranted to determine whether these chemical modifications can modulate the bioactivity and stability of OsDT11, OsDSSR1, OsEPF1, OsRALF45/OsRALF46, OsCEP8 and OsPSY8 and as-yet-uncharacterized drought-responsive small signaling peptides, thereby improving drought resilience in rice plants (Figure 2D). Taken together, targeted engineering of the promoters of genes encoding drought-responsive small signaling peptides or of their core sequence motifs represents a promising strategy for enhancing their agricultural utility.

### 3.6. Biomanufacturing with Lower Price and High Throughput

Solid-phase peptide synthesis remains the predominant approach for producing small signaling peptides; however, its widespread application is constrained by high production costs and the use of hazardous chemical reagents [75]. Consequently, scalable and cost-efficient biomanufacturing platforms are essential to enable the broader production and application of small signaling peptides in fundamental research and agricultural applications. Prokaryotic expression systems have been successfully established for the high-yield, low-cost production of plant-derived signaling peptides [43,96,97]. Continued optimization of both prokaryotic and eukaryotic expression platforms through synthetic biology is expected to accelerate the agricultural deployment of native and engineered small signaling peptides at a lower cost and with higher throughput, thus enhancing their agricultural application to promote drought stress tolerance in rice plants (Figure 2E).

### 3.7. Field Application and Safety Assessment

A major limitation is the absence of studies evaluating the long-term effects of OsDT11, OsDSSR1, OsEPF1, OsRALF45/OsRALF46, OsCEP8 and OsPSY8 peptides on rice plants during the reproductive phase and yield losses under drought stress [62,63,64,65,66,67]. Before the widespread deployment of synthetic OsDT11, OsDSSR1, OsEPF1, OsRALF45/OsRALF46, OsCEP8 and OsPSY8 peptides to enhance drought tolerance in rice plants, rigorous field trials are required to determine their long-term efficacy, ecological safety, potential detrimental effects on human health and sustainability, together with multi-year evaluations of agronomic performance (Figure 2F). Hence, future investigations should prioritize field-based validation across diverse rice genotypes, drought intensities, and developmental stages. For the agricultural application of exogenous small signaling peptides, their bioavailability and uptake, stability in soil and on leaf surfaces, and application dosage per hectare require careful evaluation. Additionally, their economic cost should also be assessed relative to those of other drought-tolerance biostimulants, for instance, phytohormones, γ-aminobutyric acid, flavonoids, and melatonin. Given the potential for trans-kingdom mobility of small signaling peptides [98], comprehensive ecological surveillance is also necessary to identify possible off-target effects on soil microbiome composition and function, as well as on non-target organisms. Furthermore, the potential adverse or pleiotropic effects of these small signaling peptides on rice growth and development should be systematically assessed (Figure 2F). Regulatory heterogeneity constitutes a significant barrier to the commercialization of small signaling peptides. Accordingly, regulatory frameworks should be developed with due consideration of public concerns and long-term risk assessments.

In conclusion, the identification of previously uncharacterized drought-responsive small signaling peptides and the clarification of their mechanistic signaling pathways would prominently expedite the genetic development of drought-resistant rice varieties, thereby promoting sustainable agricultural practices in the face of fluctuating climatic conditions.

## Data Availability

No new data were created or analyzed in this study. Data sharing is not applicable to this article.

## References

[1] Nyasulu M., Medison R.G., Mushtaq W., Mataka S.N. (2026). Stress-tolerance genes in rice: Bridging gene discovery, functional validation, and breeding applications. Front. Plant Sci..

[2] Tomar A., Kumar C., Parmar K., Khan N., Singh R., Dwivedi S.K., Prasad D. (2025). Innovative metabolic reprogramming in rice: Unlocking drought resilience through microbial consortia interaction and sustainable agriculture. 3 Biotech.

[3] Hassan M.A., Dahu N., Hongning T., Qian Z., Yueming Y., Yiru L., Shimei W. (2023). Drought stress in rice: Morpho-physiological and molecular responses and marker-assisted breeding. Front. Plant Sci..

[4] Meng Q., Zhang N., Yuan Z., Yan Y., Lv X., Zhang Y., Liu L., Zhang J., Zhang H. (2026). Signal transduction and regulatory networks of the rice root system under soil water deficit. Plant Physiol. Biochem..

[5] Geng A., Lian W., Wang Y., Liu M., Zhang Y., Wang X., Chen G. (2024). Molecular mechanisms and regulatory pathways underlying drought stress response in rice. Int. J. Mol. Sci..

[6] Hu F.W., Qin B.Y., Lin J.J., Chen T.T., Li S.F., Xiong J., Fu G.F. (2025). Unraveling the nexus of drought stress and rice physiology: Mechanisms, mitigation, and sustainable cultivation. Plant Stress.

[7] Caine R.S., Yin X., Sloan J., Harrison E.L., Mohammed U., Fulton T., Biswal A.K., Dionora J., Chater C.C., Coe R.A. (2019). Rice with reduced stomatal density conserves water and has improved drought tolerance under future climate conditions. New Phytol..

[8] Fatima P., Singh S., Mishra A., Kumar R., Singh A.M., Parveen S., Kumari M., Dei S., Shamim M.d. (2026). Morphophysiological hormonal and molecular mechanisms of rice adaptation to drought and salinity stress. Discov. Plants.

[9] Fan X., Zhang Y., Gu P., Naz M. (2025). Epitranscriptomic control of drought tolerance in rice: The role of RNA methylation. Plants.

[10] Gong N., Han Y., Wang Z., Wang Y., Du Y., Chen Z., Sun H., Li J. (2025). Research progress on genes and regulatory mechanisms of drought resistance in rice. Plant Sci..

[11] Liu Q., Dong Z., Han X., Cao J., Hu C., Deng X., Zhou L., Wu L., Yu X., You C. (2025). Photosynthetic physiological mechanisms of leaf and sheath organs to drought stress in water-saving and drought-resistant Rice. Physiol. Plant.

[12] Luo L.J. (2010). Breeding for water-saving and drought-resistance rice (WDR) in China. J. Exp. Bot..

[13] Zhang Z., Han H., Zhao J., Liu Z., Deng L., Wu L., Niu J., Guo Y., Wang G., Gou X. (2025). Peptide hormones in plants. Mol. Hortic..

[14] Ji C., Li H., Zhang Z., Peng S., Liu J., Zhou Y., Yang Y., Han H. (2025). The power of small signaling peptides in crop and horticultural plants. Crop J..

[15] Xiao F., Zhou H., Lin H. (2025). Decoding small peptides: Regulators of plant growth and stress resilience. J. Integr. Plant Biol..

[16] Álvarez-Urdiola R., Riechmann J.L. (2025). The ’non-conventional’ peptidome: A new layer in plant regulatory mechanisms. Plant Commun..

[17] Chen H., Yu J., Li W.X. (2023). Non-conventional peptides in plants: From gene regulation to crop improvement. Crop J..

[18] Jia M., Yang H., Li B., Xie Z., Cao S., Pan L., Zhang B., Zhou Y. (2026). Root development progression involves RALF peptide-mediated ROS signaling and pectin dynamics in rice. Nat. Commun..

[19] Xiao W., Zhang N., Wen D., Qi Y., Xing J., Peng Y., Wang L. (2026). The OsRALF5-FLR1 module regulates **rice** root development and provides a target for studying cell-cell communication. Rice.

[20] Kim H.J., Kim J.H., Kim E.J., Son Y.J., Hong W.J., Kim D.E., Je B.I., Jung K.H., Kim Y.J. (2026). Pollen-specific OsRALF5 regulates pollen tube growth in *Oryza sativa*. J. Plant Biol..

[21] Kwon O.K., Jeong A.R., Park C.J. (2026). OsRALF26 serves as an endogenous **signal** recognised by XA21 to promote robust and distal resistance in rice. Plant Biotechnol. J..

[22] Kwon O.K., Moon H., Jeong A.R., Yeom G., Park C.J. (2024). Rice small secreted peptide, OsRALF26, recognized by FERONIA-Like Receptor 1 induces immunity in rice and Arabidopsis. Plant J..

[23] Kwon O.K., Jeong A.R., Moon H., Shin R., Park C.J. (2025). Cross-species induction of plant immunity by oryza-specific small secreted peptide, OsRALF26. Rice Sci..

[24] Kim E.J., Kim J.H., Hong W.J., Kim E.Y., Kim M.H., Lee S.K., Min C.W., Kim S.T., Park S.K., Jung K.H. (2023). Rice pollen-specific OsRALF17 and OsRALF19 are essential for pollen tube growth. J. Integr. Plant Biol..

[25] Kim J.H., Son Y.J., Kim E.J., Jung K.H., Kim Y.J. (2025). Pollen tube-expressed RUPO forms a complex with OsMTD2 and OsRALF17 and OsRALF19 peptides in rice (*Oryza sativa*). J. Plant Physiol..

[26] Chu H., Qian Q., Liang W., Yin C., Tan H., Yao X., Yuan Z., Yang J., Huang H., Luo D. (2006). The floral organ number 4 gene encoding a putative ortholog of Arabidopsis CLAVATA3 regulates apical meristem size in rice. Plant Physiol..

[27] Suzaki T., Yoshida A., Hirano H.Y. (2008). Functional diversification of CLAVATA3-related CLE proteins in meristem maintenance in rice. Plant Cell.

[28] Suzaki T., Toriba T., Fujimoto M., Tsutsumi N., Kitano H., Hirano H.Y. (2006). Conservation and diversification of meristem maintenance mechanism in Oryza sativa: Function of the FLORAL ORGAN NUMBER 2 gene. Plant Cell Physiol..

[29] Xu W., Tao J., Chen M., Dreni L., Luo Z., Hu Y., Liang W., Zhang D. (2017). Interactions between FLORAL ORGAN NUMBER 4 and floral homeotic genes in regulating rice flower development. J. Exp. Bot..

[30] Ren D., Xu Q., Qiu Z., Cui Y., Zhou T., Zeng D., Guo L., Qian Q. (2019). FON4 prevents the multi-floret spikelet in rice. Plant Biotechnol. J..

[31] Ohmori Y., Tanaka W., Kojima M., Sakakibara H., Hirano H.Y. (2013). *WUSCHEL-RELATED HOMEOBOX4* is involved in meristem maintenance and is negatively regulated by the CLE gene *FCP1* in rice. Plant Cell.

[32] Chu H., Liang W., Li J., Hong F., Wu Y., Wang L., Wang J., Wu P., Liu C., Zhang Q. (2013). A CLE-WOX signalling module regulates root meristem maintenance and vascular tissue development in rice. J. Exp. Bot..

[33] Suzaki T., Ohneda M., Toriba T., Yoshida A., Hirano H.Y. (2009). FON2 SPARE 1 redundantly regulates floral meristem maintenance with FLORAL ORGAN NUMBER2 in rice. PLoS Genet..

[34] Xiang D., Meng F., Wang A., Wu Y., Wang Z., Zheng S., Mao C. (2021). Root-secreted peptide OsPEP1 regulates primary root elongation in rice. Plant J..

[35] Wang J., Li Y., Li M., Zhang W., Lu Y., Hua K., Ling X., Chen T., Guo D., Yang Y. (2024). Translatome and transcriptome analyses reveal the mechanism that underlies the enhancement of salt stress by the small peptide Ospep5 in plants. J. Agric. Food Chem..

[36] Zhang B., Xin B., Sun X., Chao D., Zheng H., Peng L., Chen X., Zhang L., Yu J., Ma D. (2024). Small peptide signaling via OsCIF1/2 mediates Casparian strip formation at the root endodermal and nonendodermal cell layers in rice. Plant Cell.

[37] Sui Z., Wang T., Li H., Zhang M., Li Y., Xu R., Xing G., Ni Z., Xin M. (2016). Overexpression of peptide-encoding *OsCEP6.1* results in pleiotropic effects on growth in rice (*O. sativa*). Front. Plant Sci..

[38] Lu L., Wang Y., Huang Z., Qiu S., Lin J., Feng Y., Zhang Y., Chen X., Xie S., Ma Y. (2025). A small peptide miPEP172b encoded by primary transcript of miR172b regulates salt tolerance in rice. Plant Physiol. Biochem..

[39] Lu L., Chen X., Chen J., Zhang Z., Zhang Z., Sun Y., Wang Y., Xie S., Ma Y., Song Y. (2024). MicroRNA-encoded regulatory peptides modulate cadmium tolerance and accumulation in rice. Plant Cell Environ..

[40] Xia J., Yamaji N., Ma J.F. (2013). A plasma membrane-localized small peptide is involved in rice aluminum tolerance. Plant J..

[41] Wang P., Yao S., Kosami K.I., Guo T., Li J., Zhang Y., Fukao Y., Kaneko-Kawano T., Zhang H., She Y.M. (2020). Identification of endogenous small peptides involved in rice immunity through transcriptomics- and proteomics-based screening. Plant Biotechnol. J..

[42] Wang P., Jia H., Guo T., Zhang Y., Wang W., Nishimura H., Li Z., Kawano Y. (2023). The secreted immune response peptide 1 functions as a phytocytokine in rice immunity. J. Exp. Bot..

[43] Chen X., Liang F., Yao H., Zhao Y., Shu D., Zhang Y., Shen J., Cai Y. (2026). Identification and characterization of OsSSIP, a conserved immune peptide regulating rice defense. Plant Physiol..

[44] Liu M., Zhang G., Zeng L., Yan J., Xin P., Chu J., Wang Y., Wang Q., Wang S. (2026). Pathogen-induced endogenous small peptide phytosulfokine perceived by the membrane receptor OsPSKR2 enhances disease resistance in rice. Plant Cell Environ..

[45] Harshith C.Y., Palchaudhuri D., Dey R., Raju S., Shivaprasad P.V. (2025). OsWRKY53 dictates wound responses in rice through fine-tuning crosstalk between PEP and PSK-mediated signalling. Plant J..

[46] Harshith C.Y., Pal A., Chakraborty M., Nair A., Raju S., Shivaprasad P.V. (2024). Wound-induced small-peptide-mediated signaling cascade, regulated by OsPSKR, dictates balance between growth and defense in rice. Cell Rep..

[47] Jin J., Hua L., Zhu Z., Tan L., Zhao X., Zhang W., Liu F., Fu Y., Cai H., Sun X. (2016). GAD1 Encodes a Secreted Peptide That Regulates Grain Number, Grain Length, and Awn Development in Rice Domestication. Plant Cell.

[48] Xiong L., Huang Y., Liu Z., Li C., Yu H., Shahid M.Q., Lin Y., Qiao X., Xiao J., Gray J.E. (2022). Small EPIDERMAL PATTERNING FACTOR-LIKE2 peptides regulate awn development in rice. Plant Physiol..

[49] Jin J., Xiong L., Gray J.E., Hu B., Chu C. (2023). Two awn-development-related peptides, GAD1 and OsEPFL2, promote seed dispersal and germination in rice. Mol. Plant.

[50] Guo T., Lu Z.Q., Xiong Y., Shan J.X., Ye W.W., Dong N.Q., Kan Y., Yang Y.B., Zhao H.Y., Yu H.X. (2023). Optimization of rice panicle architecture by specifically suppressing ligand-receptor pairs. Nat. Commun..

[51] Zhang D., Zhu Q., Qin P., Yu L., Li W., Sun H. (2025). The Peptide-encoding *CLE25* gene modulates drought response in Cotton. Agriculture.

[52] Lim C.W., Bae Y., Kim D.S., Lee S.C. (2026). Identification and functional analysis of CLE genes associated with drought tolerance in pepper. Plant Sci..

[53] Wu X., Tao S., Wang Z., Sun T., Zhang Z., Yang S., Xu M., Huang C., Wang S., Ke X. (2026). A CLE11b-CLE16 signaling relay mediates root-shoot-root crosstalk for drought adaptation in common bean. Adv. Sci..

[54] Lu S., Wang T., Yang P., Chang S., Yang Z., Xiao F. (2026). A genome-wide atlas of small secreted peptides (SSPs) in cotton identifies GDRP as a novel peptide hormone-enhancing drought tolerance via ABA and MAPK signaling. Plant J..

[55] Xu K., Tian D., Wang T., Zhang A., Elsadek M.A.Y., Liu W., Chen L., Guo Y. (2023). Small secreted peptides (SSPs) in tomato and their potential roles in drought stress response. Mol. Hortic..

[56] Yang W., Li R., Feng M., Qin Z., Zhang Y., Peng H., Yao Y., Hu Z., Ni Z., Song W. (2025). The TaCEP15 peptide signaling cascade modulates primary root length and drought tolerance in wheat. Sci. Adv..

[57] Jiao Z., Han S., Li Z., Huang M., Niu M.X., Yu X., Liu C., Wang H.L., Yin W., Xia X. (2022). *PdEPFL6* reduces stomatal density to improve drought tolerance in poplar. Ind. Crops Prod..

[58] He S., Sun H., Chen Q., Yang Y., Zhou Z., Chang S., Lu S., Liang Z., Yang J., Fei X. (2025). Comprehensive identification of cotton EPF/EPFL receptors and functional characterization of the GhEPFL1-1-GhER1 module in drought tolerance. BMC Plant Biol..

[59] Qiao R., Yang J., Deng Y., Duan X., Li X., Zhu F., Liu M., Mou J., Zhang N., Si H. (2024). Genome-wide identification of Epidermal Pattern Factor (EPF) gene family in potato and functional characterization of *StEPF4* in regulating drought stress. Agronomy.

[60] Xia H., Wang Q., Chen Z., Sun X., Zhao F., Zhang D., Fei J., Zhao R., Yin Y. (2024). Identification and functional analysis of the EPF/EPFL gene family in maize (*Zea mays* L.): Implications for drought stress response. Agronomy.

[61] Zhang X., Ghishkori Z.G.N., Hussain I., Javaid M.H., Zhu G., Huang J., Gulzar R.M.A. (2026). Genome-wide identification of *PSK* gene family and effects of abscisic acid (ABA) in regulating antioxidant activity and ROS signaling under drought stress in *Brassica napus*. Int. J. Mol. Sci..

[62] Li X., Han H., Chen M., Yang W., Liu L., Li N., Ding X., Chu Z. (2017). Overexpression of OsDT11, which encodes a novel cysteine-rich peptide, enhances drought tolerance and increases ABA concentration in rice. Plant Mol. Biol..

[63] Cui Y., Li M., Yin X., Song S., Xu G., Wang M., Li C., Peng C., Xia X. (2018). OsDSSR1, a novel small peptide, enhances drought tolerance in transgenic rice. Plant Sci..

[64] Mohammed U., Caine R.S., Atkinson J.A., Harrison E.L., Wells D., Chater C.C., Gray J.E., Swarup R., Murchie E.H. (2019). Rice plants overexpressing *OsEPF1* show reduced stomatal density and increased root cortical aerenchyma formation. Sci. Rep..

[65] Jing X.Q., Shi P.T., Zhang R., Zhou M.R., Shalmani A., Wang G.F., Liu W.T., Li W.Q., Chen K.M. (2024). Rice kinase OsMRLK63 contributes to drought tolerance by regulating reactive oxygen species production. Plant Physiol..

[66] Aggarwal S., Rathore R.S., Rakhi R., Kumari S., Singla-Pareek S.L., Mustafiz A. (2025). *OsCEP8*-mediated abiotic stress response is associated with auxin and sugar homeostasis in plants. Environ. Exp. Bot..

[67] Shim Y., Rim E.Y., Liao J.C., Cho M.J., Austin G., Carlos P.W., Kulkarni S.S., Payne R.J., Ercoli M.F., Ronald P.C. (2026). Signaling by a tyrosine-sulfated peptide balances growth and osmotic stress response in rice. Proc. Natl. Acad. Sci. USA.

[68] Liu L., Liu X., Bai Z., Tanveer M., Zhang Y., Chen W., Shabala S., Huang L. (2024). Small but powerful: RALF peptides in plant adaptive and developmental responses. Plant Sci..

[69] Ali B., Khan Z., Imin N. (2026). CEP-CEPR1 signalling in cereal crops: Integrating nitrogen demand, root system architecture, and drought resilience. Plant Cell Environ..

[70] Taleski M., Jin M., Chapman K., Taylor K., Winning C., Frank M., Imin N., Djordjevic M.A. (2024). CEP hormones at the nexus of nutrient acquisition and allocation, root development, and plant-microbe interactions. J. Exp. Bot..

[71] Aggarwal S., Kumar A., Jain M., Sudan J., Singh K., Kumari S., Mustafiz A. (2020). C-terminally encoded peptides (CEPs) are potential mediators of abiotic stress response in plants. Physiol. Mol. Biol. Plants.

[72] Chen L., Brun P., Buri P., Fatichi S., Gessler A., McCarthy M.J., Pellicciotti F., Stocker B., Karger D.N. (2025). Global increase in the occurrence and impact of multiyear droughts. Science.

[73] Deng Q., Sharretts T., Ali T., Ao Y.Z., Chiarelli D.D., Demeke B., Marston L., Mehta P., Mekonnen M., Rulli M.C. (2025). Deepening water scarcity in breadbasket nations. Nat. Commun..

[74] Panda D., Mishra S.S., Behera P.K. (2021). Drought tolerance in rice: Focus on recent mechanisms and approaches. Rice Sci..

[75] Yu X., Liu J., Wang W., Shen J., Shi K., Li J.F., Ye J., Rhodes J., Zipfel C., Li C. (2025). Plant-derived peptides: From identification to agronomic applications. Mol. Plant.

[76] Li Z., Jin J., Wang Y., Long W., Ding Y., Hu H., Wei L. (2023). Example: Explainable deep learning framework for the prediction of plant small secreted peptides. Bioinformatics.

[77] Wang R., Zhou Z., Wu X., Jiang X., Zhuo L., Liu M., Li H., Fu X., Yao X. (2024). An Effective plant small secretory peptide recognition model based on feature correction strategy. J. Chem. Inf. Model..

[78] Takahashi F., Suzuki T., Osakabe Y., Betsuyaku S., Kondo Y., Dohmae N., Fukuda H., Yamaguchi-Shinozaki K., Shinozaki K. (2018). A small peptide modulates stomatal control via abscisic acid in long-distance signalling. Nature.

[79] Yang W., Feng M., Yu K., Cao J., Cui G., Zhang Y., Peng H., Yao Y., Hu Z., Ni Z. (2025). The TaCLE24b peptide signaling cascade modulates lateral root development and drought tolerance in wheat. Nat. Commun..

[80] Zhang L., Shi X., Zhang Y., Wang J., Yang J., Ishida T., Jiang W., Han X., Kang J., Wang X. (2019). CLE9 peptide-induced stomatal closure is mediated by abscisic acid, hydrogen peroxide, and nitric oxide in Arabidopsis thaliana. Plant Cell Environ..

[81] Shimotohno A., Matsubayashi Y., Du Y., Senthilvadivel V., Suzuki T., Kato H., Kesherwani M., Kono M., Mishiro-Sato E., Kano K. (2025). Local peptide signalling induces stomatal closure under drought stress. Nat. Commun..

[82] Li S.T., Ke Y., Zhu Y., Zhu T.Y., Huang H., Li L., Hou Z., Zhang X., Li Y., Liu C. (2024). Mass spectrometry-based proteomic landscape of rice reveals a post-transcriptional regulatory role of N^6^-ethyladenosine. Nat. Plants.

[83] Bao H., Zhou F., Ma G., Zheng G. (2025). A systematic review of single-cell RNA sequencing applications and insights in *Oryza sativa* response to environmental stresses. Mol. Biol. Rep..

[84] Wang X., Huang H., Jiang S., Kang J., Li D., Wang K., Xie S., Tong C., Liu C., Hu G. (2025). A single-cell multi-omics atlas of rice. Nature.

[85] Liu G., Lin Q., Jin S., Gao C. (2022). The CRISPR-Cas toolbox and gene editing technologies. Mol. Cell.

[86] Shi L., Li S., Zhu R., Lu C., Xu X., Li C., Huang X., Zhao X., Mao F., Li K. (2025). CRISPRepi: A multi-omic atlas for CRISPR-based epigenome editing. Nucleic Acids Res..

[87] Gaillochet C., Develtere W., Jacobs T.B. (2021). CRISPR screens in plants: Approaches, guidelines, and future prospects. Plant Cell.

[88] Zhang Z., Li X., Li J., Liu Q., Li W., Liu J., Liu D., Wang L., Yan Z., Fu P. (2026). metaRLK 2.0: An updated database of plant receptor-like kinases developed with structure- and deep learning-based functional annotation and classification. Plant Commun..

[89] Sandoval P.J., Santiago J. (2020). *In Vitro* analytical approaches to study plant ligand-receptor interactions. Plant Physiol..

[90] Sawa S., Kinoshita A., Betsuyaku S., Fukuda H. (2008). A large family of genes that share homology with CLE domain in Arabidopsis and rice. Plant Signal Behav..

[91] Zhang Y., Yang S., Song Y., Wang J. (2014). Genome-wide characterization, expression and functional analysis of CLV3/ESR gene family in tomato. BMC Genom..

[92] Wu B., Luo H., Chen Z., Amin B., Yang M., Li Z., Wu S., Salmen S.H., Alharbi S.A., Fang Z. (2024). Rice promoter editing: An efficient genetic improvement strategy. Rice.

[93] Liu L., Gallagher J., Arevalo E.D., Chen R., Skopelitis T., Wu Q., Bartlett M., Jackson D. (2021). Enhancing grain-yield-related traits by CRISPR-Cas9 promoter editing of maize CLE genes. Nat. Plants.

[94] Hu Z., Zhang L., Han H., Wang G. (2026). Engineered peptides bolster plant plasticity. J. Integr. Plant Biol..

[95] Conibear A.C. (2020). Deciphering protein post-translational modifications using chemical biology tools. Nat. Rev. Chem..

[96] Li J., Huang Y., Yu X., Wu Q., Man X., Diao Z., You H., Shen J., Cai Y. (2024). Identification and application of CLE peptides for drought resistance in solanaceae crops. J. Agric. Food Chem..

[97] Zhang L., Gleason C. (2020). Enhancing potato resistance against root-knot nematodes using a plant-defence elicitor delivered by bacteria. Nat. Plants.

[98] Tao S., Wu X., Zhang Z., Xu P. (2026). Molecular dialogue across kingdoms: The role of trans-kingdom peptides in plant-associated interactions. Plant Cell Environ..

